# The Impact of Place-Based Approaches Addressing Mental Health and Substance Use Among Adolescents: A Systematic Review of the Literature

**DOI:** 10.3389/phrs.2024.1607955

**Published:** 2025-02-14

**Authors:** Nirandeep Rehill, Kristoffer Halvorsrud, Jenny Shand, Peter Fonagy, Rosalind Raine

**Affiliations:** ^1^ Department of Primary Care and Population Health, University College London, London, United Kingdom; ^2^ Department of Clinical, Educational and Health Psychology, University College London, London, United Kingdom; ^3^ Division of Psychiatry and Language Sciences, University College London, London, United Kingdom

**Keywords:** adolescent, mental health, place-based, substance use, public health

## Abstract

**Objectives:**

We systematically appraised peer reviewed evidence assessing the impact of “place-based approaches” (PBAs) – those requiring multi-sectoral action within localities to address complex health challenges – on mental health outcomes among adolescents.

**Methods:**

We searched six databases from inception to May 2023. We defined PBAs as at least two sectors (e.g., local government, health) working collaboratively within a locality. Studies reporting mental health and substance-use among young people (aged 10–24) were included. Two authors independently assessed study quality using MMAT. Heterogeneity in PBAs, study design and outcomes prevented meta-analysis; results were narratively synthesised.

**Results:**

Thirty-three publications presented data from 22 PBA evaluations; 6 evaluations assessed mental health or wellbeing, 16 appraised substance use. Higher quality evaluations found no impact on mental health outcomes (n = 4), and some evidence for delayed initiation (n = 4) and reduced point-in-time use (n = 10) of alcohol. Evidence for impact on binge-drinking and drug use was mixed.

**Conclusion:**

Based on very few published studies of mixed quality, PBAs have not improved mental health or wellbeing among adolescents. More evidence exists to suggest PBAs can improve certain alcohol use outcomes in young people.

## Introduction

Globally, mental health difficulties among adolescents – including depressive symptoms, self-harm, emotional and behavioural difficulties – have been increasing over the past two decades [1, 2]. Since three-quarters of adult mental disorders manifest by the age of 24 years [3], there are substantial implications for the health system, societies and economies [4]. Interventions to support adolescents’ mental health have traditionally focussed on reactive, treatment-based care; a more preventative approach addressing the social determinants that contribute to poor mental health [5, 6], may offer the greatest potential for achieving a population-wide reduction in mental health problems [7].

Health policymakers are becoming increasingly interested in working collaboratively with other sectors to address complex health challenges as they are experienced in a geographic space. Often referred to as a “Place-Based Approach” (PBA) [8], the emphasis on a defined locality [9] distinguishes this from other forms of collaboration. Within the UK context, public health authorities have emphasised the critical role of a “*joined-up place-based approach in reducing health inequalities, utilising local leadership, expertise and levers to affect this environment*” [10], while healthcare reorganisation has introduced place-based partnerships [11] focussed on improving access, experience and outcomes of health services with “*ambitions for broader coalitions with community partners influencing the wider determinants of health*.” In Australia, “Stronger Places, Stronger People” [12] focusses on the underlying disadvantage affecting some of the most deprived communities across the country.

This notion of stakeholders committing to work together to improve health, and address underlying social problems in a geography, is not new – area-based initiatives such as Health Action Zones in 1990s UK [13] and the Collective Impact model in the United States [14], are just two examples of previous schemes with the same premise of co-ordinating efforts to address specific health challenges within particular geographical contexts. Place-based interventions - the preventative changes actioned–have been varied, with some addressing structural or economic rejuvenation (e.g., high street regeneration), some emphasising community empowerment and agency (e.g., through community organising), and yet others prioritising physical environment improvements (e.g., access to green space) [9]. Multiple rationales presented for how a place-based approach might achieve stated aims include enhancing preventative approaches, increased tailoring of services to local community need, devolving power to front-line staff and the public, and improving service coordination and integration [15].

For the challenge of burgeoning adolescent mental health, there is strong evidence that the health of adolescents is affected by “proximal” determinants—the circumstances in which people are born, grow, live, work, and age [16]. In theory, therefore, action to address these circumstances in the localities in which they arise may help address the root causes of poor mental health, as well as other adolescent health outcomes. However, there is a need to unpick the evidence that already exists, from the breadth and extent of previous practice, to better understand the mechanisms by which PBAs might address the current burgeoning adolescent mental health crisis.

While some reviews have explored the impact of collaborative place-based approaches on general health outcomes in single country settings [17] or on the mental health of general populations [18, 19], none have focussed on the mental health of adolescent populations specifically. We therefore conducted the first (to our knowledge) systematic review of the evidence for collaborative place-based policy and practice approaches impacting the mental health of adolescents. We aimed to assess which place-based strategies have been evaluated in relation to their impacts on adolescent mental health or substance use outcomes, and what impacts have been demonstrated, with a focus on high-income country contexts.

Our objectives were to identify:

1. The evidence for the impact of place-based approaches (PBAs) on mental health and substance use.
2. The time periods over which impacts were measured including whether there are indications that any effects endure over time.
3. The recency of the literature evaluating these outcomes.
4. The features of place-based methods that have been evaluated in the context of adolescent mental health.

## Methods

Our review protocol was registered with PROSPERO (CRD42023461818), and we report the review following PRISMA guidelines (see Supplementary Data Sheet 1).

### Search Strategy

Our comprehensive strategy searched databases across the fields of medicine and nursing, public health, and the social sciences, namely, MEDLINE (Ovid), EMBASE, PsychInfo, Social Science Citation Index (Web of Science), Applied Social Sciences Index and Abstracts (ASSIA; ProQuest), and Cumulative Index of Nursing and Allied Health Literature (CINAHL; EBSCOhost). Searches were conducted in May 2023 with no restriction on publication date. English-language filters were applied, and only studies undertaken in Organisation for Economic Co-operation and Development (OECD; www.oecd.org) countries were included to focus on high-income contexts comparable to the United Kingdom.

We derived search strings for population (adolescents), intervention (“place-based”), and outcomes (substance use or mental health outcomes), using free text search terms and indexing terms where applicable, combined using AND. For full search terms, see Supplementary Data Sheet 2.

We also screened reference lists of included articles and used Scopus to search for papers citing the included articles to identify citations not captured by our search terms.

### Study Selection

Records were retrieved in Endnote, and duplicates removed. Inclusion and exclusion criteria are specified in Table 1. We defined adolescents as those aged 10–24 years to capture the critical transition to adulthood [20]. Citations were transferred to Rayyan (www.rayyan.ai) for title and abstract screening; all were screened by NR, with a random subset of 10% independently screened by KH. Any disparities were resolved through discussion.

**TABLE 1:** Inclusion and exclusion criteria (The impact of place-based approaches addressing mental health and substance use among adolescents, systematic review, OECD countries, to 2023).

	Inclusion	Exclusion
Population	Adolescents, defined as aged 10–24, i.e., at least 50% of population for whom outcome data is presented fall within this age bracket	Only measured MH outcomes in adolescent co-researchers (e.g., in context of youth participatory action research)
Place-based Approach	any policy, programme or intervention that fulfilled all of the following: (i) involved at least two sectors (e.g., health, local government, voluntary and community sector, commerce) where at least one sector was health or local government operating within the locality, OR was an urban renewal project. (ii) designed/adapted to meet needs within a specific local geography (e.g., neighbourhood, rural area, town, city or conurbation) (iii) focussed on addressing key elements of place: the physical, social or economic environment (iv) assessed impacts on mental health, wellbeing or associated constructs (e.g., self-efficacy, loneliness, emotional adaptation) or substance misuse	Any policy, programme or intervention (i) implemented over a geographical area larger than a conurbation, with no adaptation at more local levels. (ii) Focused on an emergency context, e.g., in a conflict or disaster zone (iii) Focused on an exclusively virtual or digital community (iv) Focussed on treatment of mental illness without addressing the physical social or economic environment (v) Focussed on individual behaviour change (so 1:1 education for individuals excluded but universal group-based family- school- or community-focussed education programmes included)
Language	Publications printed in English	
Publication Date	Any	
Geographical Location	OECD countries	
Research Type	Primary studies of any evaluation design	Cross-sectional/cohort studies not evaluating a policy, programme or intervention (e.g., those exploring risk factors only) Publications not presenting empirical findings
Publication Type	Peer reviewed journal articles, conference abstracts	Editorials, discussion papers, opinion pieces, letters and commentaries, dissertations

Full-text articles were assessed in Endnote independently by NR and KH, with disagreements resolved through discussion.

### Data Extraction and Quality Assessment

NR extracted key information into an Excel spreadsheet for analysis, including details about context (setting, geography, years implemented), place-based approach (process, interventions, description of theory of change), and study (design, mental health and substance use outcomes at each timepoint, duration of follow-up). Quality assessment of each included study was conducted independently by NR and KH using the Mixed Methods Assessment Tool [21], chosen for its flexibility across a range of study designs, and disagreements discussed until consensus was reached. To aid the synthesis of impacts, we classified evaluations as high, medium, or low quality based on meeting 4, 2 to 3, or 1 quality assessment criteria, respectively. We synthesised data from higher quality studies (i.e., high and medium-quality) first in order to state observed associations, and then noted alignment with findings from lower-quality studies subsequently.

### Data Synthesis

Given the diversity of outcome metrics, evaluation timepoints, and study designs, we used a narrative synthesis review method [22] to synthesise findings. Outcomes were summarised for each PBA evaluation rather than for each publication. This avoided “double counting” where studies reported on a cohort at multiple intervals yet retained information on how impacts changed over time. Impacts and study quality were tabulated by outcome type (mental health or substance use) and study design (randomised, non-randomised with comparators, or before-and-after studies with no comparator) to aid synthesis across similar designs. Finally, we used concept-mapping to inductively identify features common to more than one PBA, to discern any patterns that might elucidate the impacts of PBAs on outcomes [22].

## Results

**FIGURE 1:**
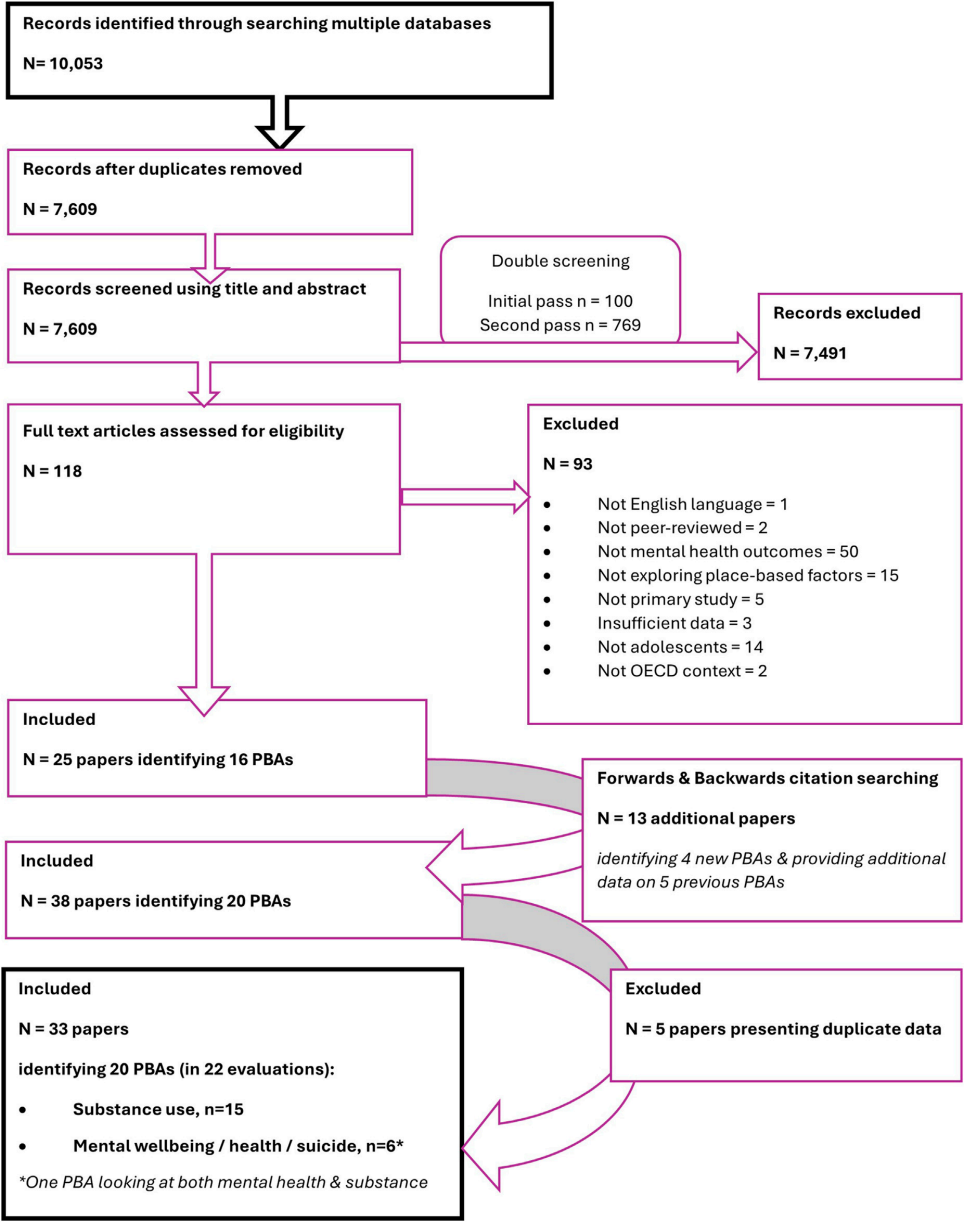
Literature search and screening process (The impact of place-based approaches addressing mental health and substance use among adolescents, systematic review, OECD countries, to 2023).

We assessed titles and abstracts of 7,609 records against inclusion and exclusion criteria, reviewed full texts of 118 publications and included 25 publications investigating 16 PBAs. Forward and backward citation searching from these articles revealed another 13 publications. Exclusion of 5 publications with duplicate data resulted in 33 publications included in the review (Figure 1).

### Publication Characteristics

Thirty-three included publications reported data from 22 evaluations of 20 PBAs and employed a range of designs, from RCTs to cross-sectional studies (Table 2). The PBAs evaluated were implemented over four decades from 1980. The majority of publications focussed on substance use outcomes (n = 28). Only six examined the impacts of PBAs on mental health outcomes; four were of PBAs implemented since 2007, suggesting interest in PBAs to address mental health is a more recent trend than for substance use.

**TABLE 2:** Included studies and place-based approaches: overview (The impact of place-based approaches addressing mental health and substance use among adolescents, systematic review, OECD countries, to 2023).

Publication Characteristics^a^	Category	Mental health publications (n = 6)	Substance use publications (n = 28)
Study design	RCT	2	15
	Non-randomised study with comparators regions	—	8
	Before and after study	1	5
	Analytic Cross-sectional study	1	—
	Qualitative/Mixed Methods	2	—
Outcome	Mental wellbeing	2	—
	Mental ill-health or suicide	5	—
	Substance use: alcohol only	—	11
	Substance use: alcohol and drugs	—	17

PBA Evaluations	Category	Mental health PBAs (n = 6)	Substance use PBAs (n = 16)
Country	United States	2	10
	Sweden	1	2
	United Kingdom	2	—
	Iceland	—	1
	Australia	—	1
	Canada	1	—
	Spain	—	1
	Netherlands	—	1
Year implementation started	pre 1990	1	2
	1991–2000	0	6
	2001–2010	2	6
	2011–2020	3	2
Duration of follow-up	0–1 year	2	1
	>1 – 3 years	3	4
	>3–5 years	—	5
	>5 years	1	6

^a^ One publication looked at both mental ill-health and substance use outcomes, while another looked at both mental ill-health and mental wellbeing.

Three PBAs were evaluated in multiple publications; PROmoting School-Community-University Partnerships to Enhance Resilience (PROSPER) [23–26]; Communities That Care [27–34], and The Icelandic Prevention Model [35, 36]. For these, we synthesise and present analyses per PBA (for key features) or PBA evaluation (for impact measures), rather than per publication, to avoid double-counting.

### Overview of the PBAs

Publications did not always describe the PBAs in detail. One-third did not make explicit a theory of how the approach would lead to intended outcomes. Therefore, it was not possible to develop a unifying theory of change as recommended by Popay et al [22].

Where PBAs were described, detail typically focussed on either the process of collaboration, planning and intervention selection – *process* - or the more specific *interventions* themselves. Several PBAs followed a pre-defined framework, usually designed by academic collaborators, and implemented in several geographies simultaneously. For example, in PROSPER [23] university-based prevention co-ordinators supported local multi-stakeholder community teams to select and implement at scale school-based and family-based programs from a relatively narrow list of evidence-based interventions, with strict monitoring of fidelity and minimal adaptation. In contrast, in Well London [37], local stakeholders identified multiply-deprived neighbourhoods where a core volunteer team used community cafes and community-based participatory approaches to design with community members the ‘best-practice’ intervention that they thought would benefit their area the most. Interventions were not detailed in evaluation papers but ranged from changes to the physical environment (e.g., green spaces), health promotion activities (e.g., mental wellbeing promotion), social activities (e.g., community food growing); how these were implemented was locally adapted in discussion with residents and based on existing local assets.

Mapping key PBA features (Table 3) identified the following as commonalities across multiple approaches, although none were universal to all:

**TABLE 3:** Key features of place-based approaches (The impact of place-based approaches addressing mental health and substance use among adolescents, systematic review, OECD countries, to 2023).

Target outcome	Place-based approach	Process						Interventions implemented: Target and type							
		External framework		Needs assessment		Local input		Young people and their families				Pathway		Suppliers	Not known
		Menu of interventions^a^	External technical support^b^	Epidemiological	Participatory	Local design of strategies^c^	Community organising^d^	In schools	For parents/Families	Youth development	Promotions^e^	Outreach	Referral pathways	All Supply-side^f^	Not specified
Mental health	Comprehensive public health activities [42]					High	x	x	x	x		x		x	x
	London 2012 Olympics and Legacy Masterplan [55]					Unclear									x
	Agenda Gap [53]					Unclear				x					
	Systems change for suicide prevention [54]					Unclear		x			x		x		x
	Well London [37]	x	x		x	High									x
Mental health and Substance use	Communities That Care [27, 28, 29, 30, 31, 32, 33, 34, 52]	x	x	x		Mid		x	x	x					
Substance use	Strategic Prevention Framework [38]	x	x	x		High	x	x	x					x	
	Alcohol, less is better [41]					Mid		x			x			x	x
	Community Interventions on an American Indian Reservation [51]		x			Low		x	x	x	x				
	The Swedish six-community prevention trial [39]	x	x			Low		x	x		x			x	x
	Beat da Binge [44]				x	Mid				x	x				
	Alcohol moderation in the Achterhoek [40]	x				Mid		x	x	Focus	x			x	
	Icelandic Model of Adolescent Substance Use Prevention [35, 36]		x	x		High		x	x	x					
	A school/family/community prevention partnership [45]					High	x	x	x	x	x			x	
	Project Northland [46]					Mid	x	x	x	x	x			x	
	Day One Community Partnership [47]					Mid	x	x	x	x	x			x	
	Neighbourhoods Engaging with Students [48]					Mid		x			x			x	x
	PROSPER [23–26]	x	x			Low		x	x						
	Trelleborg Project [49]			x		Mid		x	x					x	x
	Communities Mobilizing for Change on Alcohol [50]					Mid	x				x				x

^a^ Interventions selected from menu of evidence-based or best practice interventions.

^b^ Technical support [e.g., training/consultations/advice] provided by external programme advisors.

^c^ Extent to which strategies were designed by local collaboration.

^d^ Extent to which local communities and non-statutory organisations were engaged to implement the desired interventions.

^e^ Promotional activities, e.g., awareness events, marketing, media.

^f^ Addressed supply of alcohol to young people, for example, through a) training and education for retailers, responsible beverage schemes; b) increased local law enforcement, e.g., compliance checks, monitoring of drug trafficking; c) city policy to regulate availability, or advocacy for such policy.

#### Providing a Menu of Evidence-Based Interventions, With Implementation Support

Six of the 20 PBAs used pre-determined “menus” of evidence-based or best-practice interventions from which to select interventions to implement within places [23, 27, 37–40], although there was insufficient detail to understand how evidence-base had been appraised in these cases. These six PBAs also provided local places with external technical support such as training to enable implementation of the interventions within their locality.

#### Adapting Strategy to Local Needs and Context

Ensuring that interventions delivered were matched to local needs, context and assets was core to some PBAs. This was achieved by incorporating at least one of the following:

A. A collaborative coalition of multiple local stakeholders designing the local approach to meet locally-identified need and/or local priorities. Fourteen PBAs described this as a key process [27, 35–38, 40–50].
B. A community organising implementation approach, where local community stakeholders, such as voluntary organisations or local businesses, were engaged to support putting plans into action. Six PBAs had this feature [38, 42, 45–47, 50].

PBAs with a menu of interventions as described in 1 above tended to place less emphasis on adapting to local context as described in 2, although the Strategic Prevention Framework [38] and Well London [37] attempted a similar emphasis on both elements.

#### Focussing on Individual-Level Change or Environmental Change, or Both

Interventions implemented across reviewed PBAs intervened at individuals, family and community or environmental socio-ecologic levels (Table 3). PBAs predominantly focussing on building skills and resilience among youth were common, comprising seven of 16 substance use studies [23, 27, 35, 36, 44, 51, 52] and four of six mental health studies [32, 42, 53, 54]. Some substance use PBAs addressed the supply environment in addition to targeting young people, for example, by training suppliers or voluntary responsible supplier schemes, increasing law enforcement by compliance checks on alcohol retailers or drug trafficking monitoring, or extending existing legislation limiting supply to minors [38–41, 45–49]. One study examined the impact of improved access to urban green space and amenities on mental health, with no individual-level interventions incorporated [55].

### Quality Assessment

Two-thirds of evaluations were of high or medium quality (see Supplementary Data Sheet 3). Of the six high quality evaluations, one examined mental health [55], four examined substance use [27–29, 40, 41], and one examined both [31].

### PBAs Assessing Mental Health and Wellbeing (n = 6)

Only six PBAs reported on mental health and wellbeing outcomes (Table 4). Two aimed to improve mental health within a locality [53, 54], two aimed to improve a broad range of wellbeing outcomes [37, 42], and two measured mental wellbeing as a secondary or indirect outcome [32, 55]. The diverse interventions evaluated spanned regeneration associated with the London Olympics [55], neighbourhood community development [37], building resilience among youths in school and community [32], community youth health promotion [42], developing youth mental health policy collaborators [53], and a suicide prevention programme for at-risk youth [54].

**TABLE 4:** Study results by outcome type: Mental health outcomes and substance use outcomes (The impact of place-based approaches addressing mental health and substance use among adolescents, systematic review, OECD countries, to 2023).

Outcome: Mental Health (MH) or Substance Use (SU)	Study design	Place-base approach evaluation; Publication(s)	Outcomes assessed	Follow-up intervals	Narrative description: Effect of PBA Changes reported as statistically significant based on 95% confidence intervals or probability of *p* = 0.05 are described	Study Quality^a^
MH	Cluster RCT	Well London; [37]	Psychological Distress	2 years	No clear effect of intervention on psychological distress at 2 years	Medium
MH	Cluster RCT	Communities That Care (trial) [32]	Major depressive disorder at ages 19,21 or 23 Generalised anxiety disorder at ages 19,21 or 23	9, 10 and 12 years	No clear effect of intervention on incidence of major depressive disorder or generalised anxiety after 12th grade	Medium
MH	Non-randomised	London 2012 Olympics and Legacy Masterplan; [55]	Become depressed No longer depressed Remained depressed Wellbeing	6 months 18 months	No clear effect of intervention on becoming depressed Adolescents depressed at baseline were 53% more likely to no longer be depressed in intervention borough at 6 months, but this was not sustained at 18 months Adolescents depressed at baseline were 78% more likely to remain depressed at 6 months in intervention borough, this increased to 93% at 18 months No clear effect on wellbeing at either timepoint	High
MH	Before and after, mixed methods	Agenda Gap; [53]	Self-efficacy Resilience Other (qual study)	9 months	No change in self efficacy or resilience at 9 months Qualitative study suggested that shifts resulted from Agenda Gap at the individual, family, and community level, including reconceptualization of mental health, expanded social awareness and agency, and increased capacity for influencing systems change to promote positive mental health and wellbeing	High
MH	Before and after, mixed methods	Systems change for suicide prevention; [54]	Suicide rates among young adults aged 18–24 Suicide attempt in past 12 months Suicidal ideation	3 years 2 years 2 years	Suicide rates among 18–24 year olds decreased by 76% in the intervention area, and 50% and 66% in comparator areas Over 2 years suicide attempts in past 12 months decreased by 1.2% and suicidal ideation decreased by 5.9%	Low
MH	Analytic cross-sectional	Comprehensive public health activities; [42]	“Depressed” “Have had suicidal thoughts”	n/a^b^	Proportion stating they were “depressed” was 7% lower in intervention area (*p* < 0.01) Proportion stating they “have had suicidal thoughts” was 8%–10% lower in intervention area (*p* < 0.01)	Low
SU	RCT	Communities That Care (trial); [66], [27–34]	Incidence of alcohol initiation Alcohol use in the last 30 days Binge drinking in the last 2 weeks Sustained abstinence from alcohol, any drug use, or gateway substance use Prevalence of 30 days use – inhalants, marijuana, illicit drugs	4 years 6 years 8 years 10 years 12 years	Longitudinal cohort analyses: Alcohol initiation was reduced in intervention areas: At 4 years, students in control communities were 60% more likely to initiate the use of alcohol between grade 7 and grade 8 than students in CTC communities. At 6 years, students in intervention communities were 38% less likely to initiate the use of alcohol in grade 10 than students in control communities. At 8 years students in CTC communities were 31% more likely than students in control communities to have abstained from alcohol. This effect was not clearly sustained in a subsample at 10 years but was observed at 12 years follow-up Alcohol use: At 4 years: students in control communities had a 25% higher likelihood of having used alcohol in the last 30 days, and a 40% higher likelihood of binge drinking in the past 2 weeks, compared to intervention communities. The impact was greater for male youths than females. This difference was not observed from 6 years of follow-up Abstinence from Drugs: At 8 years, students in CTC communities were 32% more likely than students in control communities to have abstained from grade 5 through to grade 12. This effect was not sustained in the subsample at years 9–12 follow-up. At 10 years: students in CTC communities were 49% more likely than students in control communities to have abstained from gateway substances (alcohol cigarettes and marijuana combined) from grade 5 through to grade 12 Prevalence of 30 days use of marijuana, inhalants or other illicit drugs: There was no clear difference between intervention and control communities at 4 years or any subsequent timepoint Cross-sectional analyses: When comparing cross-sectional surveys of students over time, no difference in alcohol or drug use was detected in 6th, 8th, or 10th graders, 4–6 years after implementation (Rhew 2016) Effect of exposure to CTC: Results from inverse probability weighted multilevel models indicated larger effects in 10th grade (6 years follow-up) for youth who remained in their study community for the first 2 years of CTC intervention implementation compared to ITT estimates (Rhew 2018)	Hawkins and Rhew: high Oesterle and Kuklinski: medium
SU	RCT	Project Northland; [46]	Tendency to Use Alcohol Past month alcohol use Binge drinking	7 years Phase 1: years 1–3; Phase 2: years 5–7	During Phase 1: Students in the intervention schools were significantly less likely than students in the reference schools to increase their Tendency to Use Alcohol (*p* < 0.01), past month alcohol use (*p* < 0.01) and binge drinking (*p* < 0.01) During interim phase Students in the intervention schools were significantly more likely to increase their Tendency to Use Alcohol (*p* = 0.03), past month alcohol use (*p* < 0.01) and binge drinking (*p* = 0.04) During Phase 2: Students in the intervention schools were significantly less likely to increase their Tendency to Use Alcohol (*p* = 0.03) and binge drinking (*p* = 0.02), and marginally (*P* = 0.07) less likely to increase past month alcohol use	Low
SU	RCT	PROSPER; [24–26, 56]	Initiation of alcohol and drug use Past month and Past year alcohol and drug use Frequency of drinking, drunkenness and drug use Lifetime use	4.5 years 6.5 years 7.5 years (to age 18) 14 years (to age 25)	Alcohol: No clear difference in alcohol-related outcomes between intervention and control areas were observed at any time point, apart from 5% lower risk of new alcohol users and 7% lower risk of new youth ever experiencing drunkenness, observed at 4.5 years; and a lower growth in drunkenness over the entire 14 year period in the intervention areas Marijuana: There were some impacts observed on marijuana use during adolescence including lower past year use at 4.5 and 6.5 years (not sustained at 7.5 or 12 years), lower frequency of marijuana use at 6.5 and 7.5 years (not sustained at 14 years), and lower lifetime use at 4.5 and 7.5 years. While this was not sustained at 14 years, growth in lifetime use across the period was significantly lower in intervention areas Past month and past year other drug use: Results showed significantly lower drug use in the intervention group at one or both time points for most outcomes at 6.5 years, across a range of substances at 7.5 years, but no clear effect for past month or year analyses at 14 years Lifetime drug use: unlike alcohol, lifetime drug use was lower across most substances at 7.5 years (from 9.4% lower for marijuana to 41% lower for methamphetamines), and remained lower at 14 years for methamphetamines (37% lower), LSD (26% lower) and non-prescription narcotics (25% lower) Growth analyses: intervention participants demonstrated slower growth on most of the outcomes examined, including lifetime use of illicit substances and prescription narcotics. Results were weaker for marijuana use and no 10- wave effects were found for alcohol use	Medium
SU	RCT	Communities Mobilizing for Change on Alcohol (CMCA); [50]	Drank alcohol in the past 30 days Episodic heavy drinking prevalence	3 years	Among 18–20 year olds: past-month alcohol use decreased 7% in the intervention versus the control communities. Among high school students there was no clear effect There was no clear effect on episodic heavy drinking in either age cohort	Medium
SU	Non-randomised	Strategic Prevention Framework; [38]	Binge drinking	4 years	No clear effect of the intervention on binge drinking	Medium
SU	Non-randomised	Alcohol, less is better; [41]	self-reported alcohol consumption	2.5 years	Reduced by 2.3 drinks per week in PBA regions compared to control regions	High
SU	Non-randomised	Community Interventions Designed to Reduce Alcohol and Substance Abuse by Young People on an American Indian Reservation; [51]	Drank alcohol in the past month; Binge drinking episode, past 2 weeks; Started getting drunk before 9th grade Used marijuana, past month; Used cocaine or crack, past year; Used inhalants, past month	4 years	Prevalence of most substance use outcomes decreased in both intervention and control areas, with no clear difference between the two The exception was inhalant use which increased in both intervention and control areas	Medium
SU	Non-randomised	Communities That Care (Pennsylvania); [52]	30 days alcohol use (%) Lifetime alcohol use (%) Recent binge drinking (%) 30 days marijuana use; Lifetime marijuana use	8 years	Odds of all substance use outcomes were lower in CTC regions. Odds of 30-day and lifetime alcohol use outcomes were 5% lower, odds of binge drinking was 6% lower, and odds of 30-day and lifetime marijuana use was 15% lower	Medium
SU	Non-randomised	The Swedish six-community alcohol and drug prevention trial; [39]	Per capita alcohol consumption Binge drinking once or more per year	4 years	No clear effect of PBA on *per capita* alcohol consumption No clear effect on binge drinking overall, although among year 9 females only, binge drinking reduced by 20% in the trial communities compared to 10% in the control communities	Low
SU	Non-randomised	Alcohol moderation among adolescents in the Achterhoek; [40]	Recent alcohol use Recent binge drinking:	1 year and 5 years Second grade and fourth grade	Second grade: After 1 year, decline in recent alcohol use was 11% stronger in the intervention region compared to reference region and remained significant after 5 years. Similarly, the decline in binge drinking was 6% stronger in the intervention region as compared to the reference region at 1 year and remained (estimated 5% stronger) after 5 years No clear difference among 4th grade students in recent alcohol use or binge drinking at 1 or 5 years	High
SU	Non-randomised	Icelandic Model of Adolescent Substance Use Prevention; [35]	Any alcohol use (30 days): Alcohol intoxication during the last 30 days	12 years	The observed reduction in both alcohol use and alcohol intoxication over the 12 years was greater in intervention areas compared to control areas	Medium
SU	Non-randomised	Neighborhoods Engaging with Students project; [48]	Heavy drinking in past 2 weeks	1 year	Over the year, the likelihood of any heavy drinking fell among students at NEST intervention schools relative to students at the comparison school (odds ratio = 0.75, *p* < 0.05) Frequency of heavy drinking among students reduced at NEST intervention schools relative to students at the comparison school (unstandardized β = −0.20, *p* < 0.05). There was no difference in the number of drinks consumed	Low
SU	Before and after	Beat da Binge; [44]	Drinks Alcohol Short-term risky drinking	14 months	No clear effect on proportion drinking alcohol, although short term risky drinking fell by 1.2%	Low
SU	Before and after	A school/family/community substance abuse prevention partnership intervention; [45]	Monthly alcohol use Lifetime alcohol use Monthly use of marijuana Lifetime use of marijuana	3 years. 7 years. 12 years 7 years, 12 years	8% decrease in monthly alcohol use over years 7–12, and a 4% decrease in lifetime alcohol use Initial 12% increase then 3% decrease in monthly marijuana use. Alongside a 15% increase then a 3% decrease for lifetime use	Medium
SU	Before and after	Icelandic Prevention Model in Tarragona City; [36]	lifetime alcohol use Lifetime intoxication Lifetime cannabis use	4 years	No clear difference in lifetime alcohol use or intoxication Lifetime cannabis use was 4% lower in 15–16 year olds 4 years after the start of the approach	Medium
SU	Before and after	Day One Community Partnership; [47]	30-day alcohol use 7-day alcohol use Lifetime alcohol use Lifetime drunkenness 30 days drunkenness	1 year 2 years 3 years (for 7th, 9th and 12 graders)	30-day alcohol use declined significantly for 7th and 12th graders (p < 0.05) from baseline to the 1-year follow-up, but by the 2-year follow-up 30-day use had increased almost to baseline levels 7-day alcohol use followed a similar pattern to 30-day alcohol use for grades 7 to 9. For grade 12 there were significant decreases in 7-day alcohol use from baseline to the 1-year (*p* < 0.01) and baseline to the 3-year (p < 0.10) follow-ups among 12th graders No clear difference at any grade or timepoint in lifetime alcohol use, other than 12th grade where lifetime alcohol increased from 69.6% at baseline to 76.3% at 3 years follow = up No clear difference in lifetime drunkenness or 30 days drunkenness at 3 years	Low
SU	Before and after	Trelleborg Project; [49]	Consumers of alcohol Adolescents reporting Excessive Drinking Heavy episodic drinking in prior month	4 years	Consumers of alcohol decreased from 81.7% to 66.8% Adolescents reporting Excessive Drinking decreased from 37.2% to 23.7% Heavy episodic drinking in prior month decreased from 44.5% to 27.5% [cf Sweden: 26.1% to 24.0%, Lund: 24.8% to 24.3%)	Medium

^a^ High = 4 MMAT criteria met; medium = 2 to 3 criteria met; low = 1 criterion met.

^b^ Analytic cross-sectional study.

#### Impacts on Mental Health

Overall, higher quality studies found no improvement in mental health outcomes over periods lasting 6 months to 12 years [32, 37, 53, 55], while two studies reporting improvements were low quality with high risk of bias [42, 54]. Four studies reported on depression symptoms: one high-quality study had mixed conflicting results [55], two medium-quality studies found no impact [32, 37], and one low-quality study found some improvement [42]. One medium-quality study found no change in generalised anxiety disorder after earlier exposure to Communities That Care [32]. Two low-quality studies looked at suicide-related outcomes: one found that suicide rates, attempts, and ideation reduced over time [54], the other found that ideation was lower than in the comparator region [42].

Two high quality studies looked at wellbeing outcomes. One found no impact on wellbeing [55], while the other found no quantitative change in self-efficacy or resilience at 9 months but some qualitative evidence of improved agency [53].

Observations typically occurred within 3 years. The only study to examine impacts over a longer timeframe, an RCT, found that exposure to Communities That Care in adolescence had no impact on depression or anxiety at ages 19, 21, or 23 [32].

We focussed on high and medium quality studies only to observe relationships between PBA features and impacts for all outcomes. Studies exploring mental health outcomes were too few in number, and demonstrated too limited impact, to make any meaningful inferences.

### PBAs Assessing Substance Use (n = 16)

In contrast to the approaches assessing mental health outcomes, all PBAs assessing substance use explicitly aimed to reduce substance use, and all but one study [41] targeted adolescents or youth aged up to 25. Some PBAs focused exclusively on alcohol. Results have been examined separately for alcohol and other substances. Study findings are presented in Table 4.

#### Impacts on Binge Drinking

Binge drinking was the most commonly assessed substance use outcome, evaluated in 14 studies. Reported impacts on binge drinking varied across studies. Five high or medium-quality studies found positive impacts [27, 35, 40, 49, 52], while three medium-quality studies found inconsistent or no impact [23, 38, 51]. Impacts were generally modest in the highest quality studies and not sustained beyond 3 years, although one study identified a 6% lower prevalence of binge drinking in intervention areas 8 years post-implementation [52].

Among the low-quality studies, three found positive impacts [44, 46, 48], while two found no difference overall [39, 47].

There was some evidence suggesting that impacts on binge drinking might be more pronounced at younger ages. Jansen et al. [40] found a 6% greater reduction in binge drinking among second graders exposed to the intervention (mean age 13 years) but no impact for fourth graders (mean age 15 years), and Perry et al. [46] found greater reductions in grades 6–8 (typically aged 11–13) than in grades 11–12 (typically aged 16–18).

When comparing outcomes with PBA design, youth development featured in 3 out of 4 positive studies [32, 35, 52] versus 1 out of 3 null studies [51].

#### Impacts on Point-in-Time Alcohol Use

Point-in-time alcohol use typically referred to any alcohol use in the past 7 days, 2 weeks, or past month/30 days. Most studies (3 high-quality [27, 40, 41] and 5 medium-quality [35, 45, 49, 50, 52]) found impacts on point-in-time alcohol use in at least one cohort, ranging from a 7% greater decrease to 38% lower odds than in the comparator group, observed 1–5 years after implementation. Two medium-quality studies found no significant impact on point-in-time use during the initial years [23, 51]. Two low-quality studies found no sustained impact [44, 47].

Beyond 5 years follow-up, two evaluations no longer observed significant impacts on point-in-time alcohol use [24, 25, 28, 29, 56]. However, Communities That Care in Pennsylvania found 5% lower odds of alcohol use and lifetime drug and alcohol use still evident at 8 years post-intervention [52], while the Icelandic model found persistent impacts on alcohol use over a 12-year period [35].

For point-in-time alcohol use, interventions to address the supply of alcohol to young people were more likely to be a feature of PBAs with positive impacts [41, 45, 49] (present in 3 out of 6 evaluations) or mixed positive and negative impacts [40] (present in 1 out of 2 evaluations) than on PBAs with no impact (0 out of 2 evaluations). This pattern was not observed for binge drinking or other substance use metrics. In addition, encouraging youth development – such as diversionary activities or leadership opportunities – was a feature of 4 of the 6 positive studies [32, 35, 45, 52] and 1 of the 2 no impact studies [51].

#### Impacts on Point-in-Time Substance Use

Of the four high and medium-quality studies examining point-in-time substance use, one found a 15% lower 30-day marijuana use at 8 years post-real-world implementation of the Communities That Care intervention [52]. PROSPER found a risk reduction of similar magnitude at 4.5 years [23]; it was not sustained from 6.5 years, although risk reduction for less commonly used drugs (e.g., methamphetamines) persisted at later timepoints [24, 25, 56]. In contrast, the Communities That Care trial [27] and Cheadle et al. [51] found no impact on point-in-time drug use at any time point. One low-quality study identified an initial 12% increase followed by a 3% decrease in monthly marijuana use [45].

There was insufficient data to be able to link point-in-time substance use outcomes to specific PBA design features.

#### Impacts on Lifetime Use

Six studies examined lifetime alcohol use (that is, never having used alcohol in one’s lifetime); two high-quality studies [27, 52] and two medium-quality studies [45, 56] found a positive impact of PBAs on lifetime alcohol use, while one medium-quality study [36] and one low-quality study [47] found no impact. Where detected, measures of lifetime use relative to comparator areas ranged from 4% to 31% lower. Impacts were sustained for 4–8 years following programme implementation but waned over time [27, 28, 52, 56].

Four studies examined lifetime marijuana use. One high-quality and one medium-quality study found lifetime marijuana use relative to comparator areas ranged from 14% to 15% lower after 7–8 years of follow-up [52, 56], although no impact remained at 14 years [56]. Another high-quality study found no significant impact at any time point [28], and one medium-quality study found negative impacts in the short term but positive longer-term impacts [45].

Both longitudinal RCTs also examined lifetime use of other drugs, with contrasting findings. The Communities That Care study found no impact on other drug use [27–29], whereas the PROSPER study demonstrated a sustained effect in early adulthood limited to specific, lesser-used drugs: methamphetamines (37% lower), LSD (26% lower), and non-prescription narcotics (25% lower) [24].

For lifetime alcohol use, three out of four PBA evaluations demonstrating a positive impact selected interventions from an evidence-based menu [23, 27, 52], unlike the PBA demonstrating no impact [36].

## Discussion

Six evaluations of place-based approaches (PBAs) implemented between 1980 and 2020 have focussed on the impact on mental health of young people. Overall, higher-quality studies did not observe improvements in mental health outcomes over follow up periods ranging from 6 months to 12 years. Over the same period, sixteen PBAs have evaluated impact on reducing substance use among young people. The most consistent evidence for their effectiveness is in delaying initiation of alcohol use, and reducing point-in-time alcohol use, with positive effects demonstrated in the initial 5 years after programme implementation. Findings were predominantly positive, though less consistent, for short-term impacts on binge drinking, while evidence for lifetime and point-in-time drug use was mixed.

Impacts, where observed, tended to decline in magnitude after 5 years. This is unsurprising for “ever event” outcomes such as lifetime use, which in the same cohort are likely to reduce over time as they age, and similarly for point-in-time alcohol use, there may be increased societal acceptance of drinking behaviours as young adults pass the age of legal consumption. Notably, only a few studies provided follow-up beyond 5 years, and findings from later time-points are more susceptible to bias, so the body of evidence for impact at longer durations is particularly small.

The implementation of evidence-based interventions from a pre-determined “menu” was exclusive to approaches with positive impacts on lifetime alcohol use. We found some evidence supporting the role of supply-side interventions (voluntary training of suppliers, law enforcement or legislation) in reducing point-in-time alcohol use, and youth development was more likely to be a feature of PBAs with a positive impact on point-in-time alcohol and binge drinking than a feature of those with no impact. However, given the relatively small number of studies examining each specific outcome, in most cases we were not able to link PBA features to specific outcomes.

### Strengths and Limitations

To our knowledge, this is the first systematic review to assess the impact of place-based approaches—broadly defined as multi-sectoral collaborative working across sectors to address physical, social, or economic aspects within a region or locality—on adolescent mental health. “Place-based” is a term inconsistently defined in the literature, complicating the comprehensive identification of relevant papers; we mitigated this by adopting a broad definition of “place-based” and employing multiple search terms in our strategy.

A limitation of our study is that our exclusion of grey literature in order to focus on higher-quality peer-reviewed evaluations. Almost a third of studies included were judged to be of low quality; given this, it is plausible that relevant grey literature exists of equivalent quality to some included studies. Our synthesis approach prioritised findings from high and medium quality studies, so omitting low quality grey literature may not have had a significant bearing on our overall conclusions. However, it is possible that we may have missed evaluations with null findings that have not been published in peer-reviewed journals; in this event the overall positive impacts identified in this review may be optimistic. Additionally, search terms used for “substance use” were generic and did not include specific terms for substances such as “alcohol,” “marijuana” etc; however, multiple included articles exploring substance use included alcohol-specific programmes, suggesting that this has not obviously excluded studies focussing on a single substance only.

It is also possible that our focus on mental health outcomes measured among adolescents may have “selected out” certain approaches. PBAs, particularly those targeting upstream social determinants, are likely to operate through a myriad of pathways. For example, providing housing support to families living in poverty may improve adolescents’ mental health by reducing risk factors such as familial stress and domestic conflict. Improving the availability of community greenspace may enhance protective factors such as social connections at community events or increased physical activity. Evaluations of PBAs seeking “whole-of-community” benefits focussing on a wide range of outcomes (such as social connection or violence reduction), or on mental health outcomes aggregated across multiple generations, would not have been included in our review.

Misclassification of process and intervention features of PBAs included is also possible. Publications focussed on studying outcomes did not always provide detail about the approaches themselves nor analyse implementation processes, leaving a limited amount of data subject to author interpretation.

### Comparison With Other Reviews

While this is the first review to explore international evidence for the impact of PBAs on mental health, wellbeing, and substance use among adolescents within high-income contexts, other reviews have focussed on different definitions of place-based interventions, different outcomes or different populations.

A recent review by Lin et al. [17] focussed on the impact of comprehensive community initiatives (CCIs) on a wide range of health outcomes among children and young people in the USA only. This review included the Communities That Care and PROSPER RCTs from our review. Like our findings, they identified that CCIs delayed initiation of and reduced substance use across an array of substances and points in time, particularly for “hard drugs” and alcohol. They did not identify studies reporting mental health outcomes as defined in our review but found evidence that CCIs strengthen a range of protective factors and reduce risk factors in multiple contexts (e.g., peer, family, and community).

Two reviews focussed on the impact of built environment interventions on loneliness and mental health in all-age or adult populations [18, 19]. Both reviews found few studies and, like our review, found no evidence for the impact of urban regeneration. The latter review also identified literature on “urban green infrastructure,” the use of local community facilities, and active engagement in green spaces, concluding that current evidence for mental health impacts was weak.

Our review was unable to demonstrate that “collaborative strength” is relevant to implementation success in place-based approaches [57, 58]. Across included studies, the role and process of local collaboration varied from highly prescribed “top-down” approaches led or supported by external agents (e.g., university or prevention programme staff) to “home-grown” partnerships of local organisations convening as a response to locally identified challenges. However, there was insufficient commentary in most papers to appraise the success of the collaborative process. A recent umbrella review [59] synthesised systematic and scoping reviews of collaborations between healthcare (e.g., hospital and primary care) and non-healthcare (e.g., local government, housing, social services, or transportation) agencies. The authors found little convincing evidence that collaboration between these agencies improves a broad range of health outcomes, concluding that such collaborations might not work, may be very difficult to implement (identifying a range of consistently recognised barriers and facilitators), or may be challenging to evaluate.

### Why Haven’t PBAs Worked for Mental Health?

It is notable that in the context of a strong UK policy steer towards place-based working to address health inequalities and the wider determinants of health, there is a lack of evidence demonstrating place-based approaches can improve mental health and wellbeing in this age group. In contrast to multiple approaches (e.g., PROSPER and Communities That Care) that have targeted substance use at scale, equivalent frameworks have not been widely applied to address adolescent mental health within places.

One challenge may be the lack of evidence-based practices known to prevent (as opposed to treat) mental health difficulties in this age group. PBAs implementing evidence-based practices achieved greater success in preventing substance use [46], yet notably one such PBA - Communities That Care - which incorporates evidence-based practices delivered in schools to promote protective factors for youth development, such as prosocial involvement and interactions—did not reduce depression and anxiety incidence at ages 19 to 21.

A related challenge is how to evaluate a preventative mental health approach, including which outcomes to measure and when. Developing a theory of change may be one helpful tool [60]. Doherty et al [61], when designing a collaborative mental health intervention, found that a theory of change helped to confirm a shared vision in terms of long-term systems change goals, and to structure the baseline evaluation framework; however, challenges remained in developing a shared vision of *how* change occurs.

Recognising the complexity inherent in enacting change in places may require evaluations to understand the “system” as a whole, appreciating dynamic relationships between determinants when planning interventions. Conceptualising interventions as “events in systems” [62] draws from complexity science. It recognises that impact can be achieved not only through implementing evidence-based interventions at scale, which may be vulnerable to implementation failings across different contexts, but also by shifting the system itself in order to drive change.

By way of illustration, a recent paper by Cattan et al. [63], published after our searches were conducted, evaluated HeadStart, an area-based programme funding selected English local authorities to design and implement new interventions promoting young people’s mental health, wellbeing, and resilience over 6 years. The evaluators focused on absenteeism, academic attainment, and school exclusion as outcomes. They identified a transient decrease in exclusions, which they suggested might indicate a culture shift in schools towards non-exclusion rather than a change in the prevalence of externalising behaviours, given that no concurrent change in absence or attainment was observed. Using participatory systems-modelling during intervention design might highlight how exclusion culture perpetuates negative mental health (illustrated as a reinforcing feedback loop within the system), thus identifying it as an intervention target. This can focus evaluators towards firstly detecting shifts in exclusion culture in schools, prior to impacts on mental health, wellbeing, or attainment.

Finally, it is possible that PBAs make no significant difference to adolescent mental health. A critique of multi-level frameworks underpinning “place” as a specific health determining context is that they downplay the accrual of “risk” to mental health over years highlighted by a life-course approach. The implication is that place-based changes for current adolescents are too slow and too small, and population-level change in mental health requires action to address the predominant excess risk posed by disadvantage in the early years of life [64], or more fundamentally, action on the structural social determinants perpetuated through income, education and opportunity that lead to the unequal distribution of health determinants [65].

### Implications for Policy and Practice

Given the lack of demonstrated impact of these approaches on mental health outcomes, policymakers should exercise caution in expecting any improvements on observed mental health through collaborative place-based initiatives targeting local mental health determinants. Instead, greater consideration should be given to how change is expected to occur as part of intervention design; this will also support more effective monitoring and evaluation.

### Implications for Research

Future research should better articulate theories of change underpinning approaches to promote adolescent mental health and identify metrics to measure relevant change within realistic timeframes. Additionally, future evaluations may benefit from borrowing from systems science to understand where to intervene within a system, measure such shifts, map onward trajectories to impact, and identify the most relevant outcome metrics for demonstrating meaningful change within local places.
