# Supplementary material for: The Impact of Place-Based Approaches Addressing Mental Health and Substance Use Among Adolescents: A Systematic Review of the Literature
Source: Public Health Rev. 2025 Feb 14;45:1607955. doi: 10.3389/phrs.2024.1607955 (PMC11867790; doi:10.3389/phrs.2024.1607955)
Supplement: Supplementary file 1 [file DataSheet3.docx]

**Figure: Results of Quality Assessment Using MMAT Tool**

*MMAT question rating: green = yes, yellow = can’t tell, red = no*

*Overall quality: white=high quality, light grey = moderate quality, dark grey = low quality*

|  | **Outcome** | **Question stem** | **.1** | **.2** | **.3** | **.4** | **.5** |
| --- | --- | --- | --- | --- | --- | --- | --- |
| **Hawkins 2009; 2012; 2014** | **SU** | **2. Randomized controlled trials** |  |  |  |  |  |
| **Rhew 2016; 2018** | **SU** | **2. Randomized controlled trials** |  |  |  |  |  |
| **Frostick 2017** | **MH** | **2. Randomized controlled trials** |  |  |  |  |  |
| **Osterle 2010; 2015; 2018** | **SU** | **2. Randomized controlled trials** |  |  |  |  |  |
| **Spoth 2011; 2013; 2017; 2022** | **SU** | **2. Randomized controlled trials** |  |  |  |  |  |
| **Kuklinski 2021** | **SU & MH** | **2. Randomized controlled trials** |  |  |  |  |  |
| **Wagenaar 1999** | **SU** | **2. Randomized controlled trials** |  |  |  |  |  |
| **Perry 2022** | **SU** | **2. Randomized controlled trials** |  |  |  |  |  |
| **Jansen 2016** | **SU** | **3. Non-randomized studies** |  |  |  |  |  |
| **Bagnardi 2010** | **SU** | **3. Non-randomized studies** |  |  |  |  |  |
| **Anderson-Carpenter 2016** | **SU** | **3. Non-randomized studies** |  |  |  |  |  |
| **Chilenski 2019** | **SU** | **3. Non-randomized studies** |  |  |  |  |  |
| **Cheadle 1995** | **SU** | **3. Non-randomized studies** |  |  |  |  |  |
| **Kristjansson 2010** | **SU** | **3. Non-randomized studies** |  |  |  |  |  |
| **Lohrman 2005** | **SU** | **3. Non-randomized studies** |  |  |  |  |  |
| **Meyers 2023** | **SU** | **3. Non-randomized studies** |  |  |  |  |  |
| **Stafstrom 2006** | **SU** | **3. Non-randomized studies** |  |  |  |  |  |
| **Hallgren 2012** | **SU** | **3. Non-randomized studies** |  |  |  |  |  |
| **Jainullabudeen 2015** | **SU** | **3. Non-randomized studies** |  |  |  |  |  |
| **Kohlbeck 2021** | **MH** | **3. Non-randomized studies** |  |  |  |  |  |
| **Rohrbach 1997** | **SU** | **3. Non-randomized studies** |  |  |  |  |  |
| **Berg-Kelly 1997** | **MH** | **3. Non-randomized studies** |  |  |  |  |  |
| **Saltz 2009** | **SU** | **3. Non-randomized studies** |  |  |  |  |  |
| **Cummins 2018** | **MH** | **5. Mixed methods** |  |  |  |  |  |
| **Jenkins 2023** | **MH** | **5. Mixed methods** |  |  |  |  |  |
