# Supplementary material for: The Impact of Place-Based Approaches Addressing Mental Health and Substance Use Among Adolescents: A Systematic Review of the Literature
Source: Public Health Rev. 2025 Feb 14;45:1607955. doi: 10.3389/phrs.2024.1607955 (PMC11867790; doi:10.3389/phrs.2024.1607955)
Supplement: Supplementary file 2 [file DataSheet2.docx]

**Ovid MEDLINE(R) ALL <1946 to April 18, 2023>**

1 Adolescent/ or Young Adult/ 2674222

2 (teen* or youth* or adolescen* or juvenile*).ti,ab. 504800

3 (young adj2 (adult* or person* or individual* or people* or population* or man or men or wom#n)).ti,ab. 232397

4 (youngster* or first-grader* or second-grader* or third-grader* or fourth-grader* or fifth-grader* or sixth-grader* or seventh-grader* or highschool* or college*).ti,ab. 150479

5 ((secondary or high*) adj2 (school* or education)).ti,ab. 83305

6 1 or 2 or 3 or 4 or 5 3099196

7 ((Place or area or neighbourhood or community) adj2 (initiative or intervention or approach)).ti,ab. 9875

8 ((urban or neighbourhood) adj2 (renewal or regeneration)).ti,ab. 265

9 (Place#based or area#based or area#level or neighbourhood#based or neighbourhood#level or complex communit* or collective impact or community#based or community#level or Community#change or community partnership* or community development or strength##based approach or asset#based or empowered communities or system* change or regional partnership* or metropolitan partnership*).ti,ab. 8236

10 (Place#based or area#based or area#level or neighbourhood#based or neighbourhood#level or complex communit* or collective impact or community#based or community#level or Community#change or community partnership* or community development or strengths#based approach or asset#based or empowered communities or system* change or regional partnership* or metropolitan partnership*).ti,ab. 8236

11 (Health Action Zones or New Deal for Communities or Comprehensive Community Initiatives or Children?s Communities or Communities That Care or Well London or Go Well).ti,ab. 655

12 Social Planning/ or City Planning/ or Environment Design/ or Built Environment/ or Urban Renewal/ or Community Networks/ or Community Support/ or Intersectoral Collaboration/ or Public Health Practice/ or Community-Based Participatory Research/ 34814

13 (mental or Mood or Anxiety or Depress* or despair or disruptive behavio?r or antisocial behavio?r or Distress or (emotion* adj regulati*) or Isolation or Loneliness or Panic or Post?trauma* or prolonged grief or Psychiatric or Psychologic or psychoses or psychosis or PTSD or well?being or wellbeing or Resilience or resilient or social isolation or Stress*3 or worry or fear or self?harm or bereavement or sleep-wake disorder*1 or grief or poor#sleep or burnout or mania*1 or Suicide or Suicidal or traumatization or phobia*1 or impulse control or moral distress or eating disorder*1 or Coping Behaviour or Emotional adjustment or Happiness or Self-efficacy or self efficacy or Self esteem).ti,ab. 2719551

14 (substance adj (abuse or misuse)).ti,ab. 29115

15 ((psycho* or emotional* or mental) adj (distress* or stress or health or well?being or disorder or impact)).ti,ab. 290967

16 (lack* adj control).ti,ab. 262

17 *mental disorders/ or exp *anxiety disorders/ or exp *"disruptive, impulse control, and conduct disorders"/ or exp *"feeding and eating disorders"/ or exp *mood disorders/ or exp *neurodevelopmental disorders/ or exp *neurotic disorders/ or exp *personality disorders/ or exp *"schizophrenia spectrum and other psychotic disorders"/ or exp *sleep wake disorders/ or exp *substance-related disorders/ or exp *"trauma and stressor related disorders"/ or exp *Self-Injurious Behavior/ or Mental health/ or anxiety/ or depression/ or emotional regulation/ or psychological distress/ or exp Stress, Psychological/ or happiness/ or loneliness/ or self concept/ or exp body image/ or self efficacy/ or adaptation, psychological/ or emotional adjustment/ 1469153

18 7 or 8 or 9 or 10 or 11 or 12 52024

19 13 or 14 or 15 or 16 or 17 3419324

20 6 and 18 and 19 2207

21 limit 20 to english language 2142

**Embase <1974 to 2023 April 18>**

1 Adolescent/ or Young Adult/ 2088462

2 (teen* or youth* or adolescen* or juvenile*).ti,ab. 655025

3 (young adj2 (adult* or person* or individual* or people* or population* or man or men or wom#n)).ti,ab. 311700

4 (youngster* or first-grader* or second-grader* or third-grader* or fourth-grader* or fifth-grader* or sixth-grader* or seventh-grader* or highschool* or college*).ti,ab. 282000

5 ((secondary or high*) adj2 (school* or education)).ti,ab. 105769

6 1 or 2 or 3 or 4 or 5 2760945

7 ((Place or area or neighbourhood or community) adj2 (initiative or intervention or approach)).ti,ab. 12861

8 ((urban or neighbourhood) adj2 (renewal or regeneration)).ti,ab. 271

9 (Place#based or area#based or area#level or neighbourhood#based or neighbourhood#level or complex communit* or collective impact or community#based or community#level or Community#change or community partnership* or community development or strength##based approach or asset#based or empowered communities or system* change or regional partnership* or metropolitan partnership*).ti,ab. 9504

10 (Place#based or area#based or area#level or neighbourhood#based or neighbourhood#level or complex communit* or collective impact or community#based or community#level or Community#change or community partnership* or community development or strengths#based approach or asset#based or empowered communities or system* change or regional partnership* or metropolitan partnership*).ti,ab. 9504

11 (Health Action Zones or New Deal for Communities or Comprehensive Community Initiatives or Children?s Communities or Communities That Care or Well London or Go Well).ti,ab. 772

12 Social Planning/ or City Planning/ or Environment Design/ or Built Environment/ or Urban Renewal/ or Community Networks/ or Community Support/ or Intersectoral Collaboration/ or Public Health Practice/ or Community-Based Participatory Research/ 223647

13 (mental or Mood or Anxiety or Depress* or despair or disruptive behavio?r or antisocial behavio?r or Distress or (emotion* adj regulati*) or Isolation or Loneliness or Panic or Post?trauma* or prolonged grief or Psychiatric or Psychologic or psychoses or psychosis or PTSD or well?being or wellbeing or Resilience or resilient or social isolation or Stress*3 or worry or fear or self?harm or bereavement or sleep-wake disorder*1 or grief or poor#sleep or burnout or mania*1 or Suicide or Suicidal or traumatization or phobia*1 or impulse control or moral distress or eating disorder*1 or Coping Behaviour or Emotional adjustment or Happiness or Self-efficacy or self efficacy or Self esteem).ti,ab. 3465733

14 (substance adj (abuse or misuse)).ti,ab. 39953

15 ((psycho* or emotional* or mental) adj (distress* or stress or health or well?being or disorder or impact)).ti,ab. 377994

16 (lack* adj control).ti,ab. 333

17 *mental disorders/ or exp *anxiety disorders/ or exp *"disruptive, impulse control, and conduct disorders"/ or exp *"feeding and eating disorders"/ or exp *mood disorders/ or exp *neurodevelopmental disorders/ or exp *neurotic disorders/ or exp *personality disorders/ or exp *"schizophrenia spectrum and other psychotic disorders"/ or exp *sleep wake disorders/ or exp *substance-related disorders/ or exp *"trauma and stressor related disorders"/ or exp *Self-Injurious Behavior/ or Mental health/ or anxiety/ or depression/ or emotional regulation/ or psychological distress/ or exp Stress, Psychological/ or happiness/ or loneliness/ or self concept/ or exp body image/ or self efficacy/ or adaptation, psychological/ or emotional adjustment/ 2355913

18 7 or 8 or 9 or 10 or 11 or 12 243456

19 13 or 14 or 15 or 16 or 17 4578265

20 6 and 18 and 19 10416

21 exp mental health/ or *mental disease/ or exp *anxiety disorders/ or exp *behaviour disorder/ or exp *mood disorders/ or exp *autism/ or exp *Neurosis/ or exp *personality disorders/ or exp *Psychosis/ or *schizophrenia spectrum disorder/ or exp *sleep disorder/ or exp Addiction/ or *hypochondriasis/ or *anxiety/ or *depression/ or *emotion regulation/ or *mental stress/ or *happiness/ or *loneliness/ or *self concept/ or *exp body image/ or *psychological adjustment/ or wellbeing/ 1670161

23 city planning/ or neighborhood/ or exp environmental planning/ or community support/ or intersectoral collaboration/ or community program/ or social problem/ 62521

29 7 or 8 or 9 or 10 or 11 or 23 84726

30 13 or 14 or 15 or 16 or 21 4209515

31 6 and 29 and 30 5257

32 limit 31 to english language 5021

**APA PsycInfo <1806 to April Week 2 2023>**

1 Adolescent/ or Young Adult/ 0

2 (teen* or youth* or adolescen* or juvenile*).ti,ab. 364431

3 (young adj2 (adult* or person* or individual* or people* or population* or man or men or wom#n)).ti,ab. 114292

4 (youngster* or first-grader* or second-grader* or third-grader* or fourth-grader* or fifth-grader* or sixth-grader* or seventh-grader* or highschool* or college*).ti,ab. 166194

5 ((secondary or high*) adj2 (school* or education)).ti,ab. 142551

6 1 or 2 or 3 or 4 or 5 673055

7 ((Place or area or neighbourhood or community) adj2 (initiative or intervention or approach)).ti,ab. 5552

8 ((urban or neighbourhood) adj2 (renewal or regeneration)).ti,ab. 279

9 (Place#based or area#based or area#level or neighbourhood#based or neighbourhood#level or complex communit* or collective impact or community#based or community#level or Community#change or community partnership* or community development or strength##based approach or asset#based or empowered communities or system* change or regional partnership* or metropolitan partnership*).ti,ab. 5904

10 (Place#based or area#based or area#level or neighbourhood#based or neighbourhood#level or complex communit* or collective impact or community#based or community#level or Community#change or community partnership* or community development or strengths#based approach or asset#based or empowered communities or system* change or regional partnership* or metropolitan partnership*).ti,ab. 5904

11 (Health Action Zones or New Deal for Communities or Comprehensive Community Initiatives or Children?s Communities or Communities That Care or Well London or Go Well).ti,ab. 602

13 (mental or Mood or Anxiety or Depress* or despair or disruptive behavio?r or antisocial behavio?r or Distress or (emotion* adj regulati*) or Isolation or Loneliness or Panic or Post?trauma* or prolonged grief or Psychiatric or Psychologic or psychoses or psychosis or PTSD or well?being or wellbeing or Resilience or resilient or social isolation or Stress*3 or worry or fear or self?harm or bereavement or sleep-wake disorder*1 or grief or poor#sleep or burnout or mania*1 or Suicide or Suicidal or traumatization or phobia*1 or impulse control or moral distress or eating disorder*1 or Coping Behaviour or Emotional adjustment or Happiness or Self-efficacy or self efficacy or Self esteem).ti,ab. 1452753

14 (substance adj (abuse or misuse)).ti,ab. 36418

15 ((psycho* or emotional* or mental) adj (distress* or stress or health or well?being or disorder or impact)).ti,ab. 285662

16 (lack* adj control).ti,ab. 135

22 Mental health/ or Youth Mental Health/ or *mental disorders/ or exp *anxiety disorders/ or exp *bipolar disorder/ or exp *behaviour disorders/ or exp *eating disorders/ or exp *affective disorders/ or exp *Neurodevelopmental Disorders/ or exp *Neurosis/ or exp *personality disorders/ or exp *Psychosis/ or exp *sleep wake disorders/ or *illness anxiety disorder/ or *anxiety/ or *emotional regulation/ or exp *Psychological Stress/ or *happiness/ or *loneliness/ or *self-destructive behaviour/ or *self concept/ or *exp body image/ or *self efficacy/ or exp *coping behaviour/ or *emotional adjustment/ or Well Being/ or *"depression (emotion)"/ or exp *"substance related and addictive disorders"/ or exp *"stress and trauma related disorders"/ 964422

24 exp Urban Planning/ or Environmental Planning/ or Built Environment/ or exp Urban Health/ or exp Rural Health/ or Community Health/ or exp Public Mental Health/ 10953

25 7 or 8 or 9 or 10 or 11 or 24 22569

26 13 or 14 or 15 or 16 or 22 1832168

27 6 and 25 and 26 1297

28 limit 27 to english language 1247

**CINAHL**

| **#** | **Query** | **Limiters/Expanders** | **Last Run Via** | **Results** |
| --- | --- | --- | --- | --- |
| S1 | (MH Adolescent) OR (MH "Young Adult") | Search modes - Boolean/Phrase | Interface - EBSCOhost Research Databases Search Screen - Advanced Search Database - CINAHL Plus | 287,435 |
| S2 | ((TI teen* OR AB teen*) OR (TI youth* OR AB youth*) OR (TI adolescen* OR AB adolescen*) OR (TI juvenile* OR AB juvenile*)) | Search modes - Boolean/Phrase | Interface - EBSCOhost Research Databases Search Screen - Advanced Search Database - CINAHL Plus | 230,279 |
| S3 | ((TI young OR AB young) N2 ((TI adult* OR AB adult*) OR (TI person* OR AB person*) OR (TI individual* OR AB individual*) OR (TI people* OR AB people*) OR (TI population* OR AB population*) OR (TI man OR AB man) OR (TI men OR AB men) OR (TI wom?n OR AB wom?n))) | Search modes - Boolean/Phrase | Interface - EBSCOhost Research Databases Search Screen - Advanced Search Database - CINAHL Plus | 95,582 |
| S4 | ((TI youngster* OR AB youngster*) OR (TI first-grader* OR AB first-grader*) OR (TI second-grader* OR AB second-grader*) OR (TI third-grader* OR AB third-grader*) OR (TI fourth-grader* OR AB fourth-grader*) OR (TI fifth-grader* OR AB fifth-grader*) OR (TI sixth-grader* OR AB sixth-grader*) OR (TI seventh-grader* OR AB seventh-grader*) OR (TI highschool* OR AB highschool*) OR (TI college* OR AB college*)) | Search modes - Boolean/Phrase | Interface - EBSCOhost Research Databases Search Screen - Advanced Search Database - CINAHL Plus | 86,009 |
| S5 | (((TI secondary OR AB secondary) OR (TI high* OR AB high*)) N2 ((TI school* OR AB school*) OR (TI education OR AB education))) | Search modes - Boolean/Phrase | Interface - EBSCOhost Research Databases Search Screen - Advanced Search Database - CINAHL Plus | 51,565 |
| S6 | S1 OR S2 OR S3 OR S4 OR S5 | Search modes - Boolean/Phrase | Interface - EBSCOhost Research Databases Search Screen - Advanced Search Database - CINAHL Plus | 643,915 |
| S7 | (((TI Place OR AB Place) OR (TI area OR AB area) OR (TI neighbourhood OR AB neighbourhood) OR (TI community OR AB community)) N2 ((TI initiative OR AB initiative) OR (TI intervention OR AB intervention) OR (TI approach OR AB approach))) | Search modes - Boolean/Phrase | Interface - EBSCOhost Research Databases Search Screen - Advanced Search Database - CINAHL Plus | 17,156 |
| S8 | (((TI urban OR AB urban) OR (TI neighbourhood OR AB neighbourhood)) N2 ((TI renewal OR AB renewal) OR (TI regeneration OR AB regeneration))) | Search modes - Boolean/Phrase | Interface - EBSCOhost Research Databases Search Screen - Advanced Search Database - CINAHL Plus | 130 |
| S9 | ((TI Place?based OR AB Place?based) OR (TI area?based OR AB area?based) OR (TI area?level OR AB area?level) OR (TI neighbourhood?based OR AB neighbourhood?based) OR (TI neighbourhood?level OR AB neighbourhood?level) OR (TI "complex communit*" OR AB "complex communit*") OR (TI "collective impact" OR AB "collective impact") OR (TI community?based OR AB community?based) OR (TI community?level OR AB community?level) OR (TI Community?change OR AB Community?change) OR (TI "community partnership*" OR AB "community partnership*") OR (TI "community development" OR AB "community development") OR (TI "strengths?based approach" OR AB "strengths?based approach") OR (TI asset?based OR AB asset?based) OR (TI "empowered communities" OR AB "empowered communities") OR (TI "system* change" OR AB "system* change") OR (TI "regional partnership*" OR AB "regional partnership*") OR (TI "metropolitan partnership*" OR AB "metropolitan partnership*")) | Search modes - Boolean/Phrase | Interface - EBSCOhost Research Databases Search Screen - Advanced Search Database - CINAHL Plus | 4,418 |
| S10 | ((TI "Health Action Zones" OR AB "Health Action Zones") OR (TI "New Deal for Communities" OR AB "New Deal for Communities") OR (TI "Comprehensive Community Initiatives" OR AB "Comprehensive Community Initiatives") OR (TI "Children#s Communities" OR AB "Children#s Communities") OR (TI "Communities That Care" OR AB "Communities That Care") OR (TI "Well London" OR AB "Well London") OR (TI "Go Well" OR AB "Go Well")) | Search modes - Boolean/Phrase | Interface - EBSCOhost Research Databases Search Screen - Advanced Search Database - CINAHL Plus | 348 |
| S11 | (MH "Social Planning") OR (MH "City Planning") OR (MH "Environment Design") OR (MH "Built Environment") OR (MH "Urban Renewal") OR (MH "Community Networks") OR (MH "Community Support") OR (MH "Intersectoral Collaboration") OR (MH "Public Health Practice") OR (MH "Community-Based Participatory Research") | Search modes - Boolean/Phrase | Interface - EBSCOhost Research Databases Search Screen - Advanced Search Database - CINAHL Plus | 3,299 |
| S12 | ((TI mental OR AB mental) OR (TI Mood OR AB Mood) OR (TI Anxiety OR AB Anxiety) OR (TI Depress* OR AB Depress*) OR (TI despair OR AB despair) OR (TI "disruptive behavio#r" OR AB "disruptive behavio#r") OR (TI "antisocial behavio#r" OR AB "antisocial behavio#r") OR (TI Distress OR AB Distress) OR ((TI emotion* OR AB emotion*) W1 (TI regulati* OR AB regulati*)) OR (TI Isolation OR AB Isolation) OR (TI Loneliness OR AB Loneliness) OR (TI Panic OR AB Panic) OR (TI Post#trauma* OR AB Post#trauma*) OR (TI "prolonged grief" OR AB "prolonged grief") OR (TI Psychiatric OR AB Psychiatric) OR (TI Psychologic OR AB Psychologic) OR (TI psychoses OR AB psychoses) OR (TI psychosis OR AB psychosis) OR (TI PTSD OR AB PTSD) OR (TI well#being OR AB well#being) OR (TI wellbeing OR AB wellbeing) OR (TI Resilience OR AB Resilience) OR (TI resilient OR AB resilient) OR (TI "social isolation" OR AB "social isolation") OR (TI Stress*3 OR AB Stress*3) OR (TI worry OR AB worry) OR (TI fear OR AB fear) OR (TI self#harm OR AB self#harm) OR (TI bereavement OR AB bereavement) OR (TI "sleep-wake disorder*1" OR AB "sleep-wake disorder*1") OR (TI grief OR AB grief) OR (TI poor?sleep OR AB poor?sleep) OR (TI burnout OR AB burnout) OR (TI mania*1 OR AB mania*1) OR (TI Suicide OR AB Suicide) OR (TI Suicidal OR AB Suicidal) OR (TI traumatization OR AB traumatization) OR (TI phobia*1 OR AB phobia*1) OR (TI "impulse control" OR AB "impulse control") OR (TI "moral distress" OR AB "moral distress") OR (TI "eating disorder*1" OR AB "eating disorder*1") OR (TI "Coping Behaviour" OR AB "Coping Behaviour") OR (TI "Emotional adjustment" OR AB "Emotional adjustment") OR (TI Happiness OR AB Happiness) OR (TI Self-efficacy OR AB Self-efficacy) OR (TI "self efficacy" OR AB "self efficacy") OR (TI "Self esteem" OR AB "Self esteem")) | Search modes - Boolean/Phrase | Interface - EBSCOhost Research Databases Search Screen - Advanced Search Database - CINAHL Plus | 684,731 |
| S13 | ((TI substance OR AB substance) W1 ((TI abuse OR AB abuse) OR (TI misuse OR AB misuse))) | Search modes - Boolean/Phrase | Interface - EBSCOhost Research Databases Search Screen - Advanced Search Database - CINAHL Plus | 17,905 |
| S14 | (((TI psycho* OR AB psycho*) OR (TI emotional* OR AB emotional*) OR (TI mental OR AB mental)) W1 ((TI distress* OR AB distress*) OR (TI stress OR AB stress) OR (TI health OR AB health) OR (TI well#being OR AB well#being) OR (TI disorder OR AB disorder) OR (TI impact OR AB impact))) | Search modes - Boolean/Phrase | Interface - EBSCOhost Research Databases Search Screen - Advanced Search Database - CINAHL Plus | 194,687 |
| S15 | ((TI lack* OR AB lack*) W1 (TI control OR AB control)) | Search modes - Boolean/Phrase | Interface - EBSCOhost Research Databases Search Screen - Advanced Search Database - CINAHL Plus | 1,289 |
| S16 | (MM "mental disorders") OR (MM "anxiety disorders+") OR (MM "disruptive, impulse control, and conduct disorders+") OR (MM "feeding and eating disorders+") OR (MM "mood disorders+") OR (MM "neurodevelopmental disorders+") OR (MM "neurotic disorders+") OR (MM "personality disorders+") OR (MM "schizophrenia spectrum and other psychotic disorders+") OR (MM "sleep wake disorders+") OR (MM "substance-related disorders+") OR (MM "trauma and stressor related disorders+") OR (MM "Self-Injurious Behavior+") OR (MH "Mental health") OR (MH anxiety) OR (MH depression) OR (MH "emotional regulation") OR (MH "psychological distress") OR (MH "Stress, Psychological+") OR (MH happiness) OR (MH loneliness) OR (MH "self concept") OR (MH "body image+") OR (MH "self efficacy") OR (MH "adaptation, psychological") OR (MH "emotional adjustment") | Search modes - Boolean/Phrase | Interface - EBSCOhost Research Databases Search Screen - Advanced Search Database - CINAHL Plus | 443,945 |
| S17 | S7 OR S8 OR S9 OR S10 OR S11 | Expanders - Apply equivalent subjects Search modes - Boolean/Phrase | Interface - EBSCOhost Research Databases Search Screen - Advanced Search Database - CINAHL Plus | 24,404 |
| S18 | S12 OR S13 OR S14 OR S15 OR S16 | Expanders - Apply equivalent subjects Search modes - Boolean/Phrase | Interface - EBSCOhost Research Databases Search Screen - Advanced Search Database - CINAHL Plus | 865,769 |
| S19 | S6 AND S17 AND S18 | Expanders - Apply equivalent subjects Search modes - Boolean/Phrase | Interface - EBSCOhost Research Databases Search Screen - Advanced Search Database - CINAHL Plus | 1,099 |

(MH Adolescent) OR (MH "Young Adult")
((TI teen* OR AB teen*) OR (TI youth* OR AB youth*) OR (TI adolescen* OR AB adolescen*) OR (TI juvenile* OR AB juvenile*))
((TI young OR AB young) N2 ((TI adult* OR AB adult*) OR (TI person* OR AB person*) OR (TI individual* OR AB individual*) OR (TI people* OR AB people*) OR (TI population* OR AB population*) OR (TI man OR AB man) OR (TI men OR AB men) OR (TI wom?n OR AB wom?n)))
((TI youngster* OR AB youngster*) OR (TI first-grader* OR AB first-grader*) OR (TI second-grader* OR AB second-grader*) OR (TI third-grader* OR AB third-grader*) OR (TI fourth-grader* OR AB fourth-grader*) OR (TI fifth-grader* OR AB fifth-grader*) OR (TI sixth-grader* OR AB sixth-grader*) OR (TI seventh-grader* OR AB seventh-grader*) OR (TI highschool* OR AB highschool*) OR (TI college* OR AB college*))
(((TI secondary OR AB secondary) OR (TI high* OR AB high*)) N2 ((TI school* OR AB school*) OR (TI education OR AB education)))
S1 OR S2 OR S3 OR S4 OR S5
(((TI Place OR AB Place) OR (TI area OR AB area) OR (TI neighbourhood OR AB neighbourhood) OR (TI community OR AB community)) N2 ((TI initiative OR AB initiative) OR (TI intervention OR AB intervention) OR (TI approach OR AB approach)))
(((TI urban OR AB urban) OR (TI neighbourhood OR AB neighbourhood)) N2 ((TI renewal OR AB renewal) OR (TI regeneration OR AB regeneration)))
((TI Place?based OR AB Place?based) OR (TI area?based OR AB area?based) OR (TI area?level OR AB area?level) OR (TI neighbourhood?based OR AB neighbourhood?based) OR (TI neighbourhood?level OR AB neighbourhood?level) OR (TI "complex communit*" OR AB "complex communit*") OR (TI "collective impact" OR AB "collective impact") OR (TI community?based OR AB community?based) OR (TI community?level OR AB community?level) OR (TI Community?change OR AB Community?change) OR (TI "community partnership*" OR AB "community partnership*") OR (TI "community development" OR AB "community development") OR (TI "strength??based approach" OR AB "strength??based approach") OR (TI asset?based OR AB asset?based) OR (TI "empowered communities" OR AB "empowered communities") OR (TI "system* change" OR AB "system* change") OR (TI "regional partnership*" OR AB "regional partnership*") OR (TI "metropolitan partnership*" OR AB "metropolitan partnership*"))
((TI Place?based OR AB Place?based) OR (TI area?based OR AB area?based) OR (TI area?level OR AB area?level) OR (TI neighbourhood?based OR AB neighbourhood?based) OR (TI neighbourhood?level OR AB neighbourhood?level) OR (TI "complex communit*" OR AB "complex communit*") OR (TI "collective impact" OR AB "collective impact") OR (TI community?based OR AB community?based) OR (TI community?level OR AB community?level) OR (TI Community?change OR AB Community?change) OR (TI "community partnership*" OR AB "community partnership*") OR (TI "community development" OR AB "community development") OR (TI "strengths?based approach" OR AB "strengths?based approach") OR (TI asset?based OR AB asset?based) OR (TI "empowered communities" OR AB "empowered communities") OR (TI "system* change" OR AB "system* change") OR (TI "regional partnership*" OR AB "regional partnership*") OR (TI "metropolitan partnership*" OR AB "metropolitan partnership*"))
((TI "Health Action Zones" OR AB "Health Action Zones") OR (TI "New Deal for Communities" OR AB "New Deal for Communities") OR (TI "Comprehensive Community Initiatives" OR AB "Comprehensive Community Initiatives") OR (TI "Children#s Communities" OR AB "Children#s Communities") OR (TI "Communities That Care" OR AB "Communities That Care") OR (TI "Well London" OR AB "Well London") OR (TI "Go Well" OR AB "Go Well"))
(MH "Social Planning") OR (MH "City Planning") OR (MH "Environment Design") OR (MH "Built Environment") OR (MH "Urban Renewal") OR (MH "Community Networks") OR (MH "Community Support") OR (MH "Intersectoral Collaboration") OR (MH "Public Health Practice") OR (MH "Community-Based Participatory Research")
((TI mental OR AB mental) OR (TI Mood OR AB Mood) OR (TI Anxiety OR AB Anxiety) OR (TI Depress* OR AB Depress*) OR (TI despair OR AB despair) OR (TI "disruptive behavio#r" OR AB "disruptive behavio#r") OR (TI "antisocial behavio#r" OR AB "antisocial behavio#r") OR (TI Distress OR AB Distress) OR ((TI emotion* OR AB emotion*) W1 (TI regulati* OR AB regulati*)) OR (TI Isolation OR AB Isolation) OR (TI Loneliness OR AB Loneliness) OR (TI Panic OR AB Panic) OR (TI Post#trauma* OR AB Post#trauma*) OR (TI "prolonged grief" OR AB "prolonged grief") OR (TI Psychiatric OR AB Psychiatric) OR (TI Psychologic OR AB Psychologic) OR (TI psychoses OR AB psychoses) OR (TI psychosis OR AB psychosis) OR (TI PTSD OR AB PTSD) OR (TI well#being OR AB well#being) OR (TI wellbeing OR AB wellbeing) OR (TI Resilience OR AB Resilience) OR (TI resilient OR AB resilient) OR (TI "social isolation" OR AB "social isolation") OR (TI Stress*3 OR AB Stress*3) OR (TI worry OR AB worry) OR (TI fear OR AB fear) OR (TI self#harm OR AB self#harm) OR (TI bereavement OR AB bereavement) OR (TI "sleep-wake disorder*1" OR AB "sleep-wake disorder*1") OR (TI grief OR AB grief) OR (TI poor?sleep OR AB poor?sleep) OR (TI burnout OR AB burnout) OR (TI mania*1 OR AB mania*1) OR (TI Suicide OR AB Suicide) OR (TI Suicidal OR AB Suicidal) OR (TI traumatization OR AB traumatization) OR (TI phobia*1 OR AB phobia*1) OR (TI "impulse control" OR AB "impulse control") OR (TI "moral distress" OR AB "moral distress") OR (TI "eating disorder*1" OR AB "eating disorder*1") OR (TI "Coping Behaviour" OR AB "Coping Behaviour") OR (TI "Emotional adjustment" OR AB "Emotional adjustment") OR (TI Happiness OR AB Happiness) OR (TI Self-efficacy OR AB Self-efficacy) OR (TI "self efficacy" OR AB "self efficacy") OR (TI "Self esteem" OR AB "Self esteem"))
((TI substance OR AB substance) W1 ((TI abuse OR AB abuse) OR (TI misuse OR AB misuse)))
(((TI psycho* OR AB psycho*) OR (TI emotional* OR AB emotional*) OR (TI mental OR AB mental)) W1 ((TI distress* OR AB distress*) OR (TI stress OR AB stress) OR (TI health OR AB health) OR (TI well#being OR AB well#being) OR (TI disorder OR AB disorder) OR (TI impact OR AB impact)))
((TI lack* OR AB lack*) W1 (TI control OR AB control))
(MM "mental disorders") OR (MM "anxiety disorders+") OR (MM "disruptive, impulse control, and conduct disorders+") OR (MM "feeding and eating disorders+") OR (MM "mood disorders+") OR (MM "neurodevelopmental disorders+") OR (MM "neurotic disorders+") OR (MM "personality disorders+") OR (MM "schizophrenia spectrum and other psychotic disorders+") OR (MM "sleep wake disorders+") OR (MM "substance-related disorders+") OR (MM "trauma and stressor related disorders+") OR (MM "Self-Injurious Behavior+") OR (MH "Mental health") OR (MH anxiety) OR (MH depression) OR (MH "emotional regulation") OR (MH "psychological distress") OR (MH "Stress, Psychological+") OR (MH happiness) OR (MH loneliness) OR (MH "self concept") OR (MH "body image+") OR (MH "self efficacy") OR (MH "adaptation, psychological") OR (MH "emotional adjustment")
S7 OR S8 OR S9 OR S10 OR S11 OR S12
S13 OR S14 OR S15 OR S16 OR S17
S6

**ASSIA Proquest v2**

Set#: S4

Searched for: ((TI,AB(Place) OR TI,AB(area) OR TI,AB(neighbourhood) OR TI,AB(community)) NEAR/2 (TI,AB(initiative) OR TI,AB(intervention) OR TI,AB(approach))) OR ((TI,AB(urban) OR TI,AB(neighbourhood)) NEAR/2 (TI,AB(renewal) OR TI,AB(regeneration))) OR (TI,AB(Place?based) OR TI,AB(area?based) OR TI,AB(area?level) OR TI,AB(neighbourhood?based) OR TI,AB(neighbourhood?level) OR TI,AB("complex communit*") OR TI,AB("collective impact") OR TI,AB(community?based) OR TI,AB(community?level) OR TI,AB(Community?change) OR TI,AB("community partnership*") OR TI,AB("community development") OR TI,AB("strength?based approach") OR TI,AB(asset?based) OR TI,AB("empowered communities") OR TI,AB("system* change") OR TI,AB("regional partnership*") OR TI,AB("metropolitan partnership*")) OR (TI,AB(Place?based) OR TI,AB(area?based) OR TI,AB(area?level) OR TI,AB(neighbourhood?based) OR TI,AB(neighbourhood?level) OR TI,AB("complex communit*") OR TI,AB("collective impact") OR TI,AB(community?based) OR TI,AB(community?level) OR TI,AB(Community?change) OR TI,AB("community partnership*") OR TI,AB("community development") OR TI,AB("strengths?based approach") OR TI,AB(asset?based) OR TI,AB("empowered communities") OR TI,AB("system* change") OR TI,AB("regional partnership*") OR TI,AB("metropolitan partnership*")) OR (TI,AB("Health Action Zones") OR TI,AB("New Deal for Communities") OR TI,AB("Comprehensive Community Initiatives") OR TI,AB("Children?s Communities") OR TI,AB("Communities That Care") OR TI,AB("Well London") OR TI,AB("Go Well"))

Databases: Applied Social Sciences Index & Abstracts (ASSIA)

Results: 14295

Set#: S8

Searched for: (TI,AB(mental) OR TI,AB(Mood) OR TI,AB(Anxiety) OR TI,AB(Depress*) OR TI,AB(despair) OR TI,AB("disruptive behavio?r") OR TI,AB("antisocial behavio?r") OR TI,AB(Distress) OR (TI,AB(emotion*) PRE/0 TI,AB(regulati*)) OR TI,AB(Isolation) OR TI,AB(Loneliness) OR TI,AB(Panic) OR TI,AB(Post?trauma*) OR TI,AB("prolonged grief") OR TI,AB(Psychiatric) OR TI,AB(Psychologic) OR TI,AB(psychoses) OR TI,AB(psychosis) OR TI,AB(PTSD) OR TI,AB(well?being) OR TI,AB(wellbeing) OR TI,AB(Resilience) OR TI,AB(resilient) OR TI,AB("social isolation") OR TI,AB(Stress*3) OR TI,AB(worry) OR TI,AB(fear) OR TI,AB(self?harm) OR TI,AB(bereavement) OR TI,AB("sleep-wake disorder*1") OR TI,AB(grief) OR TI,AB(poor?sleep) OR TI,AB(burnout) OR TI,AB(mania*1) OR TI,AB(Suicide) OR TI,AB(Suicidal) OR TI,AB(traumatization) OR TI,AB(phobia*1) OR TI,AB("impulse control") OR TI,AB("moral distress") OR TI,AB("eating disorder*1") OR TI,AB("Coping Behaviour") OR TI,AB("Emotional adjustment") OR TI,AB(Happiness) OR TI,AB(Self-efficacy) OR TI,AB("self efficacy") OR TI,AB("Self esteem")) OR (TI,AB(substance) PRE/0 (TI,AB(abuse) OR TI,AB(misuse))) OR ((TI,AB(psycho*) OR TI,AB(emotional*) OR TI,AB(mental)) PRE/0 (TI,AB(distress*) OR TI,AB(stress) OR TI,AB(health) OR TI,AB(well?being) OR TI,AB(disorder) OR TI,AB(impact))) OR (TI,AB(lack*) PRE/0 TI,AB(control)) OR (MAINSUBJECT.EXACT.EXPLODE("Mental health") OR

MAINSUBJECT.EXACT.EXPLODE("Stress") OR

MAINSUBJECT.EXACT.EXPLODE("Anxiety") OR

MAINSUBJECT.EXACT.EXPLODE("Psychological trauma") OR

MAINSUBJECT.EXACT("Body image disturbances") OR MAINSUBJECT.EXACT.EXPLODE("Body image") OR

MAINSUBJECT.EXACT.EXPLODE("Mental depression"))

Databases: Applied Social Sciences Index & Abstracts (ASSIA)

Results: 326653

Set#: S9

Searched for: (TI,AB(teen*) OR TI,AB(youth*) OR TI,AB(adolescen*) OR TI,AB(juvenile*)) OR (TI,AB(young) NEAR/2 (TI,AB(adult*) OR TI,AB(person*) OR TI,AB(individual*) OR TI,AB(people*) OR TI,AB(population*) OR TI,AB(man) OR TI,AB(men) OR TI,AB(wom?n))) OR (TI,AB(youngster*) OR TI,AB(first-grader*) OR TI,AB(second-grader*) OR TI,AB(third-grader*) OR TI,AB(fourth-grader*) OR TI,AB(fifth-grader*) OR TI,AB(sixth-grader*) OR TI,AB(seventh-grader*) OR TI,AB(highschool*) OR TI,AB(college*)) OR ((TI,AB(secondary) OR TI,AB(high*)) NEAR/2 (TI,AB(school*) OR TI,AB(education))) OR (MAINSUBJECT.EXACT.EXPLODE("Young adults") OR

MAINSUBJECT.EXACT.EXPLODE("Adolescents"))

Databases: Applied Social Sciences Index & Abstracts (ASSIA)

Results: 185038

Set#: S10

Searched for: ((TI,AB(teen*) OR TI,AB(youth*) OR TI,AB(adolescen*) OR TI,AB(juvenile*)) OR (TI,AB(young) NEAR/2 (TI,AB(adult*) OR TI,AB(person*) OR TI,AB(individual*) OR TI,AB(people*) OR TI,AB(population*) OR TI,AB(man) OR TI,AB(men) OR TI,AB(wom?n))) OR (TI,AB(youngster*) OR TI,AB(first-grader*) OR TI,AB(second-grader*) OR TI,AB(third-grader*) OR TI,AB(fourth-grader*) OR TI,AB(fifth-grader*) OR TI,AB(sixth-grader*) OR TI,AB(seventh-grader*) OR TI,AB(highschool*) OR TI,AB(college*)) OR ((TI,AB(secondary) OR TI,AB(high*)) NEAR/2 (TI,AB(school*) OR TI,AB(education))) OR (MAINSUBJECT.EXACT.EXPLODE("Young adults") OR MAINSUBJECT.EXACT.EXPLODE("Adolescents"))) AND ((TI,AB(mental) OR TI,AB(Mood) OR TI,AB(Anxiety) OR TI,AB(Depress*) OR TI,AB(despair) OR TI,AB("disruptive behavio?r") OR TI,AB("antisocial behavio?r") OR TI,AB(Distress) OR (TI,AB(emotion*) PRE/0 TI,AB(regulati*)) OR TI,AB(Isolation) OR TI,AB(Loneliness) OR TI,AB(Panic) OR TI,AB(Post?trauma*) OR TI,AB("prolonged grief") OR TI,AB(Psychiatric) OR TI,AB(Psychologic) OR TI,AB(psychoses) OR TI,AB(psychosis) OR TI,AB(PTSD) OR TI,AB(well?being) OR TI,AB(wellbeing) OR TI,AB(Resilience) OR TI,AB(resilient) OR TI,AB("social isolation") OR TI,AB(Stress*3) OR TI,AB(worry) OR TI,AB(fear) OR TI,AB(self?harm) OR TI,AB(bereavement) OR TI,AB("sleep-wake disorder*1") OR TI,AB(grief) OR TI,AB(poor?sleep) OR TI,AB(burnout) OR TI,AB(mania*1) OR TI,AB(Suicide) OR TI,AB(Suicidal) OR TI,AB(traumatization) OR TI,AB(phobia*1) OR TI,AB("impulse control") OR TI,AB("moral distress") OR TI,AB("eating disorder*1") OR TI,AB("Coping Behaviour") OR TI,AB("Emotional adjustment") OR TI,AB(Happiness) OR TI,AB(Self-efficacy) OR TI,AB("self efficacy") OR TI,AB("Self esteem")) OR (TI,AB(substance) PRE/0 (TI,AB(abuse) OR TI,AB(misuse))) OR ((TI,AB(psycho*) OR TI,AB(emotional*) OR TI,AB(mental)) PRE/0 (TI,AB(distress*) OR TI,AB(stress) OR TI,AB(health) OR TI,AB(well?being) OR TI,AB(disorder) OR TI,AB(impact))) OR (TI,AB(lack*) PRE/0 TI,AB(control)) OR (MAINSUBJECT.EXACT.EXPLODE("Mental health") OR MAINSUBJECT.EXACT.EXPLODE("Stress") OR MAINSUBJECT.EXACT.EXPLODE("Anxiety") OR MAINSUBJECT.EXACT.EXPLODE("Psychological trauma") OR MAINSUBJECT.EXACT("Body image disturbances") OR MAINSUBJECT.EXACT.EXPLODE("Body image") OR MAINSUBJECT.EXACT.EXPLODE("Mental depression"))) AND (((TI,AB(Place) OR TI,AB(area) OR TI,AB(neighbourhood) OR TI,AB(community)) NEAR/2 (TI,AB(initiative) OR TI,AB(intervention) OR TI,AB(approach))) OR ((TI,AB(urban) OR TI,AB(neighbourhood)) NEAR/2 (TI,AB(renewal) OR TI,AB(regeneration))) OR (TI,AB(Place?based) OR TI,AB(area?based) OR TI,AB(area?level) OR TI,AB(neighbourhood?based) OR TI,AB(neighbourhood?level) OR TI,AB("complex communit*") OR TI,AB("collective impact") OR TI,AB(community?based) OR TI,AB(community?level) OR TI,AB(Community?change) OR TI,AB("community partnership*") OR TI,AB("community development") OR TI,AB("strength?based approach") OR TI,AB(asset?based) OR TI,AB("empowered communities") OR TI,AB("system* change") OR TI,AB("regional partnership*") OR TI,AB("metropolitan partnership*")) OR (TI,AB(Place?based) OR TI,AB(area?based) OR TI,AB(area?level) OR TI,AB(neighbourhood?based) OR TI,AB(neighbourhood?level) OR TI,AB("complex communit*") OR TI,AB("collective impact") OR TI,AB(community?based) OR TI,AB(community?level) OR TI,AB(Community?change) OR TI,AB("community partnership*") OR TI,AB("community development") OR TI,AB("strengths?based approach") OR TI,AB(asset?based) OR TI,AB("empowered communities") OR TI,AB("system* change") OR TI,AB("regional partnership*") OR TI,AB("metropolitan partnership*")) OR (TI,AB("Health Action Zones") OR TI,AB("New Deal for Communities") OR TI,AB("Comprehensive Community Initiatives") OR TI,AB("Children?s Communities") OR TI,AB("Communities That Care") OR TI,AB("Well London") OR TI,AB("Go Well")))

Databases: Applied Social Sciences Index & Abstracts (ASSIA)

These databases are searched for part of your query.

Results: 751

**# Web of Science Search Strategy (v0.1) - Search 2.2**

# Database: Web of Science Core Collection

# Entitlements:

- WOS.IC: 1993 to 2023

- WOS.CCR: 1985 to 2023

- WOS.SCI: 1900 to 2023

- WOS.AHCI: 1975 to 2023

- WOS.BHCI: 2005 to 2023

- WOS.BSCI: 2005 to 2023

- WOS.ESCI: 2015 to 2023

- WOS.ISTP: 1990 to 2023

- WOS.SSCI: 1900 to 2023

- WOS.ISSHP: 1990 to 2023

# Searches:

4: TS = (young NEAR/2 (adult* OR person* OR individual* OR people* OR population* OR man OR men OR wom?n )) (Topic) or ((youngster* or first-grader* or second-grader* or third-grader* or fourth-grader* or fifth-grader* or sixth-grader* or seventh-grader* or highschool* or college*)) (Topic) or ((secondary OR high*) NEAR/2 (school* OR education)) (Topic) or (teen* or youth* or adolescen* or juvenile*) (Topic)

8: TS = (Place OR area OR neighbourhood OR community ) NEAR/2 (initiative OR intervention OR approach ) (Topic) or (urban OR neighbourhood ) NEAR/2 (renewal OR regeneration) (Topic) or Place$based OR area$based OR area$level OR neighbourhood$based OR neighbourhood$level OR "complex communit*" OR "collective impact" OR Community$change OR "community partnership*" OR "community development" OR "strength$based approach" OR asset$based OR "empowered communities" OR "system* change" OR "regional partnership*" OR "metropolitan partnership*" (Topic) or "Health Action Zones" OR "New Deal for Communities" OR "Comprehensive Community Initiatives" OR "Children$s Communities" OR "Communities That Care" OR "Well London" OR "Go Well" (Topic)

12: TS=((mental OR Mood OR Anxiety OR Depress* OR despair OR "disruptive behavio$r" OR "antisocial behavio$r" OR Distress OR (emotion* NEAR/0 regulati* ) OR Isolation OR Loneliness OR Panic OR Post$trauma* OR "prolonged grief" OR Psychiatric OR Psychologic OR psychoses OR psychosis OR PTSD OR well$being OR wellbeing OR Resilience OR resilient OR "social isolation" OR Stress* OR worry OR fear OR self$harm OR bereavement OR "sleep-wake disorder$" OR grief OR poor$sleep OR burnout OR mania$ OR Suicide OR Suicidal OR traumatization OR phobia$ OR "impulse control" OR "moral distress" OR "eating disorder$" OR "Coping Behaviour" OR "Emotional adjustment" OR Happiness OR Self-efficacy OR "self efficacy" OR "Self esteem" )) OR TS=((psycho* OR emotional* OR mental ) NEAR/0 (distress* OR stress OR health OR well$being OR disorder OR impact)) OR TS=(((lack* NEAR/0 control)))

13:

#4 AND #12 AND #8
